# Chemotherapy-mediated p53-dependent DNA damage response in clear cell renal cell carcinoma: role of the mTORC1/2 and hypoxia-inducible factor pathways

**DOI:** 10.1038/cddis.2013.395

**Published:** 2013-10-17

**Authors:** J Selvarajah, K Nathawat, A Moumen, M Ashcroft, V A Carroll

**Affiliations:** 1 Division of Biomedical Sciences, St George's University of London, Cranmer Terrace, London, SW17 0RE, UK; 2 Department of Metabolism and Experimental Therapeutics, Division of Medicine, Centre for Cell Signalling and Molecular Genetics, University College London, Rayne Building, 5 University Street, London, WC1E 6JJ, UK

**Keywords:** p53, HIF-1*α*, HIF-2*α*, endothelin-1, mTORC1/2, renal cell carcinoma

## Abstract

The DNA-damaging agent camptothecin (CPT) and its analogs demonstrate clinical utility for the treatment of advanced solid tumors, and CPT-based nanopharmaceuticals are currently in clinical trials for advanced kidney cancer; however, little is known regarding the effects of CPT on hypoxia-inducible factor-2*α* (HIF-2*α*) accumulation and activity in clear cell renal cell carcinoma (ccRCC). Here we assessed the effects of CPT on the HIF/p53 pathway. CPT demonstrated striking inhibition of both HIF-1*α* and HIF-2*α* accumulation in von Hippel–Lindau (VHL)-defective ccRCC cells, but surprisingly failed to inhibit protein levels of HIF-2*α-*dependent target genes (VEGF, PAI-1, ET-1, cyclin D1). Instead, CPT induced DNA damage-dependent apoptosis that was augmented in the presence of pVHL. Further analysis revealed CPT regulated endothelin-1 (ET-1) in a p53-dependent manner: CPT increased ET-1 mRNA abundance in VHL-defective ccRCC cell lines that was significantly augmented in their VHL-expressing counterparts that displayed increased phosphorylation and accumulation of p53; p53 siRNA suppressed CPT-induced increase in ET-1 mRNA, as did an inhibitor of ataxia telangiectasia mutated (ATM) signaling, suggesting a role for ATM-dependent phosphorylation of p53 in the induction of ET-1. Finally, we demonstrate that p53 phosphorylation and accumulation is partially dependent on mTOR activity in ccRCC. Consistent with this result, pharmacological inhibition of mTORC1/2 kinase inhibited CPT-mediated ET-1 upregulation, and p53-dependent responses in ccRCC. Collectively, these data provide mechanistic insight into the action of CPT in ccRCC, identify ET-1 as a p53-regulated gene and demonstrate a requirement of mTOR for p53-mediated responses in this tumor type.

Clear cell renal cell carcinoma (ccRCC) is the most predominant type of sporadic kidney cancer, with ∼25% of patients presenting with advanced disease. Inactivating mutations in the *von Hippel–Lindau* (*VHL*) tumor-suppressor gene account for the majority of sporadic clear cell cancers.^1^ Loss of *VHL* function leads to accumulation of the *α-*subunits of hypoxia-inducible factor (HIF) transcription factors (HIF-1*α* and HIF-2*α*) that dimerize with HIF-1*β* and transactivate HIF target genes such as vascular endothelial growth factor (VEGF). Current therapies for treating metastatic ccRCC include receptor tyrosine kinase and multikinase inhibitors directed to VEGF and platelet-derived growth factor (PDGF) pathways as well as inhibitors of mammalian target of rapamycin (mTOR).^2, 3^ Despite antiangiogenic therapies having significantly increased progression-free survival in ccRCC, overall patient survival is still low as tumors eventually acquire resistance to these modalities.^4^ Therefore, combination strategies with antiangiogenics and second-generation mTOR-targeted drugs such as the dual mTOR/PI3Kinase and mTORC1/mTORC2 kinase inhibitors are currently being investigated for improved therapeutic outcome for metastatic ccRCC and other malignancies.^5^

The HIF-*α* subunits have emerged in recent years as potential therapeutic targets in ccRCC. HIF-1*α* and HIF-2*α* play a central, if complex, role in the development ccRCC. Several lines of evidence demonstrate that HIF-2*α* is the primary oncogenic driver in ccRCC.^6, 7, 8^ In addition, HIF-2*α* predominantly regulates angiogenic genes such as VEGF in this tumor type.^9, 10, 11^ In contrast, recent evidence suggests that HIF-1*α* acts as a tumor suppressor in ccRCC.^10, 12^ ccRCC is also highly resistant to chemotherapy and radiotherapy and some studies have shown that this resistance can be circumvented by inhibition of HIF-2*α*.^13, 14^ For example, previous work in pVHL-defective RCC cell lines that express constitutive HIF-2*α* has shown that ablation of HIF-2*α*, either with siRNA or by reintroduction of *VHL*, increases phosphorylation and accumulation of p53.^13, 14^ Concordantly, HIF-2*α* inhibition restored sensitivity to radiation and chemotherapy, suggesting that inhibitors of HIF-2*α* would be beneficial in combination with radiotherapy, chemotherapeutics or agents that restore p53 pathway activity. Collectively, these data have significant implications for targeting the HIF pathway directly as it still remains unclear whether inhibition of HIF-1*α* or HIF-2*α* alone or in combination would be beneficial for kidney cancer.

Camptothecin (CPT) and its analogs, topotecan and irinotecan, are topoisomerase I inhibitors that prevent topoisomerase I-mediated unwinding and DNA repair, leading to accumulation of DNA double-stranded breaks and cell death.^15^ These agents are also potent inhibitors of HIF-1*α* and have been studied extensively for HIF-1*α-*targeted therapy.^16, 17, 18, 19, 20, 21^ CPT-based nanopharmaceuticals are currently in clinical trials for advanced kidney cancer; however, to our knowledge, no reports exist as to the effects of CPT on HIF-2*α* function in ccRCC. Therefore, in this study we investigated the effects of CPT on HIF-2*α* expression and activity together with its effects on p53 accumulation and p53-dependent responses in ccRCC.

## Results

### Effect of CPT on HIF-1*α*, HIF-2*α* and HIF-*α* target genes in ccRCC

**Figure 1:**
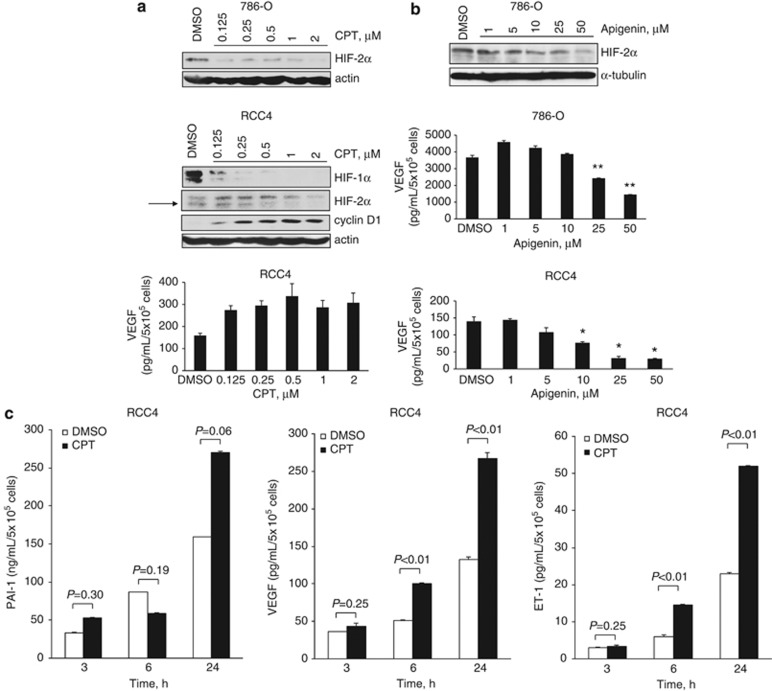
Effect of CPT and apigenin on HIF-1*α*, HIF-2*α* and HIF-*α* target genes in RCC4 and 786-O cells. (**a** and **b**) 786-O or RCC4 cells were treated with CPT or apigenin at the concentrations indicated or vehicle control (DMSO). Panels, whole-cell lysates were assayed by western blot for HIF-1*α*, HIF-2*α* and cyclin D1 proteins. Actin and/or tubulin were used as loading controls. Graphs, conditioned media were harvested after 24 h and secreted protein levels of VEGF were determined by ELISA and normalized to cell number. (**c**) RCC4 cells were treated with 2 *μ*M CPT or DMSO vehicle control for the times indicated. Conditioned media were harvested and secreted protein levels of VEGF, PAI-1 and ET-1 were determined by ELISA and normalized to cell number. Mean±S.E. of duplicate values of one representative experiment is shown. *P<0.05, **P<0.01, *t-*test compared with control

Although the inhibition of HIF-1*α* by CPT has been intensively studied, its effect on HIF-2*α* accumulation and activity in ccRCC has not, to our knowledge, been demonstrated. CPT dose dependently inhibited HIF-2*α* protein levels in VHL-defective 786-O cells expressing constitutive HIF-2*α* (Figure 1a) and HIF-1*α* and HIF-2*α* protein levels in VHL-defective RCC4 cells that express both HIF-1*α* and HIF-2*α* (Figure 1a). We next assessed the ability of CPT to inhibit a number of HIF-*α* target genes. CPT partially inhibited GLUT-1 and BNIP3 at 24 h (Supplementary Figure 1), both of which are predominantly regulated by the HIF-1*α* subunit.^11, 22^ However, despite inhibition of HIF-2*α* protein, CPT failed to have significant inhibitory activity on a number of HIF-2*α* target genes that we evaluated (Figures 1a and c and Supplementary Figure 1). Protein levels of HIF-2*α-*dependent genes, cyclin D1, VEGF, plasminogen activator inhibitor-1 (PAI-1) and endothelin-1 (ET-1), were not inhibited, but were partially elevated in RCC4 cells (Figures 1a and c). In contrast, apigenin, which is a dietary flavonoid that has been reported to inhibit HIF-1*α-* and HIF-1*α-*dependent VEGF,^23^ also inhibited HIF-2*α* protein levels and VEGF in 786-O and RCC4 cells (Figure 1b). Collectively, these data suggest that CPT is unlikely to mediate its antitumor effects through downregulation of HIF-2*α* target genes such as VEGF.

**Figure 2:**
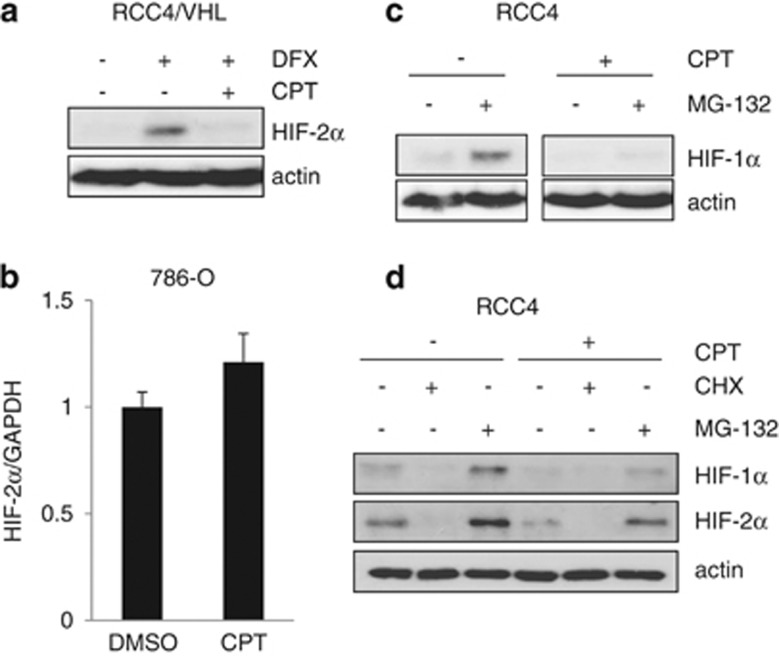
CPT inhibits HIF-1*α* and HIF-2*α* protein synthesis. (**a**) RCC4/VHL cells were incubated with 500 *μ*M desferrioxamine (DFX) in the absence or presence of 2 *μ*M CPT for 24 h as indicated. Whole-cell lysates were assayed by western blot for HIF-2*α*. Actin was used as a loading control. (**b**) The 786-O cells were incubated with 2 *μ*M CPT or vehicle control (DMSO) for 24 h and analyzed for mRNA expression of HIF-2*α* by real-time quantitative PCR relative to GAPDH. (**c** and **d**) RCC4 cells were incubated with or without 2 *μ*M CPT for 6 h in the absence or presence of 10 *μ*M MG-132 or 10 *μ*M cycloheximide (CHX) for the final 3 h as indicated. Whole-cell lysates were assayed by western blot for HIF-1*α* and HIF-2*α*. Actin was used as a loading control

We next assessed the mechanism of action of CPT on HIF-2*α* protein accumulation. Along with inhibition of constitutive HIF-2*α* protein, CPT also inhibited desferrioxamine (DFX)-induced HIF-2*α* protein accumulation in VHL-competent RCC4 cells (RCC4/VHL) (Figure 2a). CPT had no effect on HIF-2*α* mRNA levels (Figure 2b), suggesting that it did not affect HIF-2*α* mRNA synthesis or stability. As previous studies have demonstrated that CPT inhibits HIF-1*α* protein synthesis,^21^ we incubated RCC4 cells in the presence of the 26S proteasome inhibitor MG-132 in order to inhibit HIF-*α* protein degradation. CPT markedly reduced the MG-132-induced accumulation of HIF-1*α* (Figures 2c and d), consistent with previous reports.^21^ Both HIF-*α* subunits were reduced in the presence of the protein synthesis inhibitor, cycloheximide (CHX), demonstrating a requirement of protein synthesis for constitutive expression of HIF-*α* subunits (Figure 2d). CPT also inhibited HIF-2*α* in the presence of MG-132, but to a lesser extent than HIF-1*α*, suggesting that CPT also partly inhibits HIF-2*α* protein synthesis.

### CPT mediated p53 accumulation and apoptosis in ccRCC

**Figure 3:**
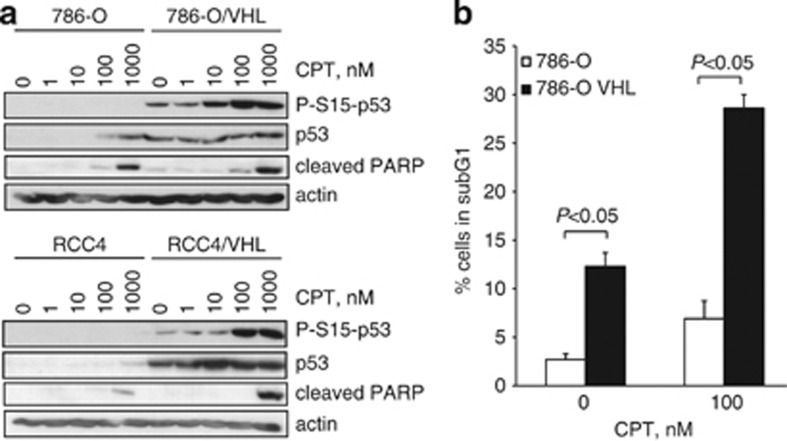
CPT-mediated increase in p53 levels and apoptosis is augmented in VHL-competent cells. (**a**) The 786-O and RCC4 cells and their VHL-expressing counterparts were treated with increasing concentrations of CPT for 24 h. Whole-cell lysates were assayed by western blot for p53 and phosphorylated-S15-p53 proteins and cleaved PARP. Actin was used as a loading control. (**b**) The 786-O and 786-O/VHL cells were treated with 100 nM CPT or vehicle for 24 h, fixed, stained with propidium iodide and the percentage (%) of cells in subG1 was assessed by flow cytometry. Mean±S.E. of three replicates is shown

We next explored the ability of CPT to induce p53 and p53-dependent responses in ccRCC cell lines. Previous studies have demonstrated increased p53 accumulation in pVHL-expressing cells.^13, 24, 25, 26^ Consistent with these studies, we found that p53 phosphorylation and accumulation was suppressed in VHL-defective 786-O cells that express HIF-2*α* (Figure 3a) and indeed RCC4 cells that express both HIF-1*α* and HIF-2*α* subunits (Figure 3a) as compared with their VHL-expressing counterparts. CPT treatment increased p53 accumulation in 786-O cells, and to a lesser extent in RCC4 cells, that was augmented in their VHL-expressing counterparts respectively. In addition, cleaved poly ADP ribose polymerase (PARP) was observed in 766-O and RCC4 cells in response to CPT that was further increased in VHL-expressing cells (Figure 3a). We next assessed the effects of CPT on sub-G1 content by flow cytometry. We found that CPT increased the percentage of cells in sub-G1 in 786-O cells that was again augmented in 786-O/VHL cells (Figure 3b and Supplementary Figure 2). Taken together, our data indicate that CPT increases p53 accumulation and apoptosis in a VHL-dependent manner. These results are consistent with previous studies that have demonstrated suppression of p53 pathway activation in VHL-defective cells.^13, 14, 24, 26^

### CPT increases ET-1 mRNA abundance in a p53-dependent manner

**Figure 4:**
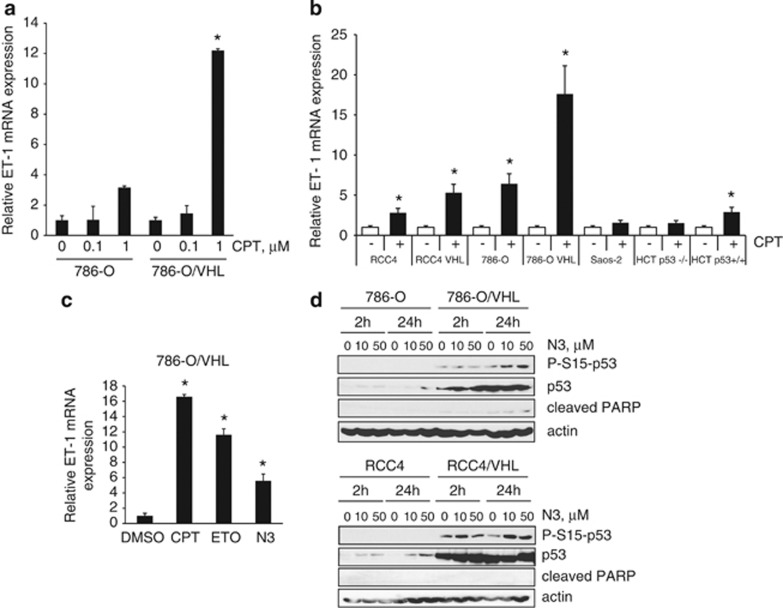
DNA-damaging agents increase ET-1 mRNA abundance in a p53-dependent manner. (**a**) The 786-O and 786-O/VHL cells were treated with increasing concentrations of CPT for 24 h and analyzed for mRNA expression of ET-1 by real-time quantitative PCR relative to GAPDH. (**b**) Cells as indicated were treated with 2 *μ*M CPT (+) or vehicle DMSO (−) for 24 h and analyzed for mRNA expression of ET-1 by real-time quantitative PCR relative to GAPDH. (**c**) The 786-O/VHL cells were treated with vehicle DMSO, 2 *μ*M CPT, 100 *μ*M etoposide (ETO) or 10 *μ*M nutlin-3a (N3) and analyzed for mRNA expression of ET-1 by real-time quantitative PCR relative to GAPDH. (**d**) The 786-O and RCC4 cells and their VHL-expressing counterparts were treated with vehicle control, 10 *μ*M or 50 *μ*M nutlin-3a (N3) for 2 or 24 h. Whole-cell lysates were assayed by western blot for phosphorylated-S15-p53, p53 and cleaved PARP proteins. Actin was used as a loading control. In the graphs, mean±S.E. of duplicate values of one representative experiment is shown. * *P*<0.05 as compared with control

In RCC4 cells we failed to see CPT-mediated inhibition of VEGF, PAI-1 and ET-1, all of which are HIF-2*α* target genes. However, we have previously demonstrated that, in addition to HIF-2*α*, VEGF protein levels are sensitive to mitogen-activated protein kinase signaling.^27^ Both extracellular regulated kinase 1/2 phosphorylation and VEGF are increased upon CPT treatment in RCC4 cells.^27^ In addition, PAI-1 is also regulated by p53.^28^ We were interested in exploring the CPT-mediated regulation of ET-1 levels further as little is known about the regulation of ET-1 in ccRCC. ET-1 mRNA levels were determined by RT-PCR. Consistent with previous reports we demonstrated that constitutive ET-1 mRNA and protein were regulated in a VHL-dependent manner, and we have previously shown that ET-1 protein levels were partly dependent on HIF-2*α* (Supplementary Figure 3).^27, 29^ Interestingly, CPT induced ET-1 mRNA abundance in a dose-dependent manner in 786-O cells that was significantly augmented following reintroduction of VHL (Figures 4a and b), suggesting a role for p53 in ET-1 induction. Similar results were observed for the RCC4 and RCC4/VHL cells: CPT increased ET-1 mRNA abundance in RCC4 cells that was further increased upon reintroduction of VHL (Figure 4b). We extended our studies to nonrenal cells and assessed the paired isogenic cell lines HCT116 p53^+/+^ and p53^−/−^ cells. We found that CPT increased ET-1 mRNA in HCT p53^+/+^ cells, but not p53^−/−^ cells, although the increase observed was not as significant as that seen in ccRCC cells (Figure 4b). No effect of CPT on ET-1 mRNA levels was observed in p53-null Saos-2 cells (Figure 4b).

**Figure 5:**
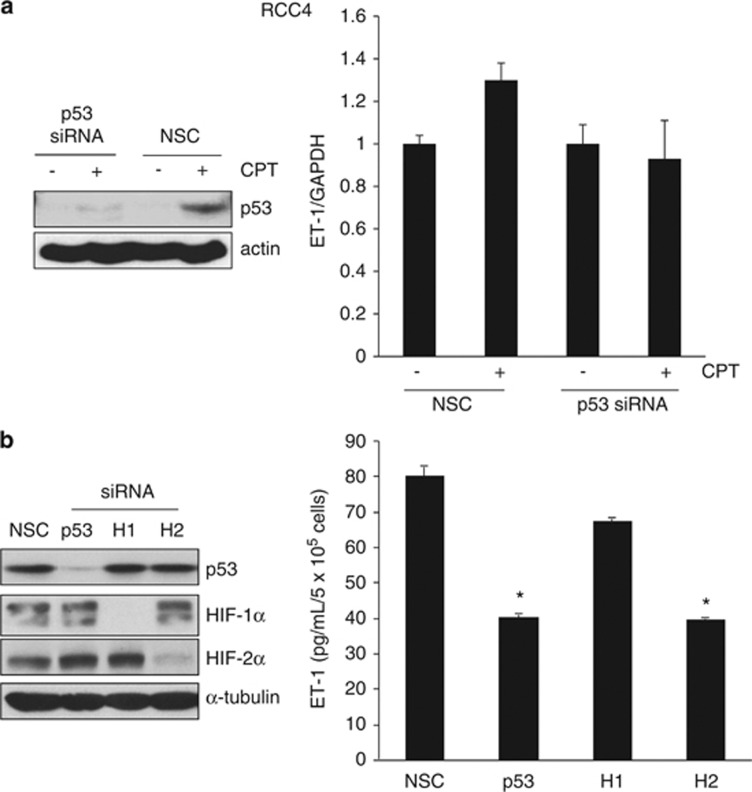
The p53-dependent regulation of ET-1 in RCC4 cells. (**a**) RCC4 cells were transfected with 10 nM siRNA to p53 or nonsilencing control (NSC) duplex for 24 h before addition of 2 *μ*M CPT (+) or DMSO vehicle control (−) for a further 24 h. Panels, whole-cell lysates were assayed by western blot for p53 protein. Actin was used as a loading control. Graph, mRNA expression of ET-1 by real-time quantitative PCR relative to GAPDH. Mean±S.E. of duplicate values of one representative experiment is shown. (**b**) RCC4 cells were transfected with 10 nM siRNA to p53, HIF-1*α* (H1), or HIF-2*α* (H2) or NSC duplex for 24 h. Panels, whole-cell lysates were assayed by western blot for p53, HIF-1*α* and HIF-2*α* proteins. Tubulin was used as a loading control. Graph, conditioned media were harvested and secreted protein levels of ET-1 were determined by ELISA and normalized to cell number. Mean±S.E. of duplicate values of one representative experiment is shown. *P<0.05, *t* test compared with control

To further assess DNA-damaging agents on ET-1 mRNA levels, we next analyzed the effects of etoposide, a topoisomerase II inhibitor. Etoposide increased ET-1 mRNA abundance in 786-O cells expressing VHL (Figure 4c). In addition, nutlin-3a, which prevents the binding of MDM2 to p53, also increased ET-1 mRNA levels (Figure 4c) as well as p53 accumulation in both 786-O and RCC4 cells that were augmented in their VHL-expressing counterparts (Figure 4d). Finally, in order to confirm that the effects of CPT on ET-1 were indeed p53 dependent, we used p53 siRNA. Downregulation of p53 with siRNA prevented the CPT-induced increase in ET-1 mRNA (Figure 5a) and inhibited ET-1 protein levels in RCC4 cells (Figure 5b). Taken together, these results demonstrate that ET-1 is induced in a p53-dependent manner in ccRCC upon DNA damage.

### p53-dependent increase in ET-1 mRNA abundance is dependent on ATM signaling

**Figure 6:**
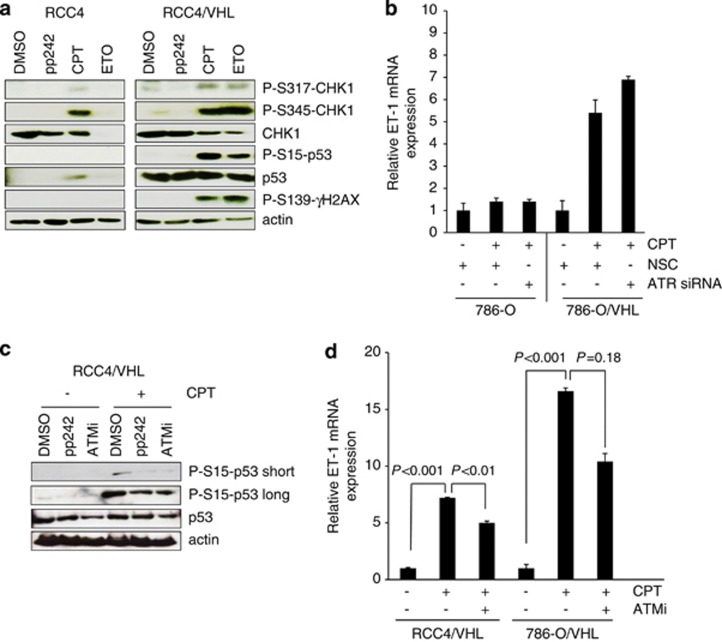
DNA damage response is suppressed in HIF-*α-*expressing cells. (**a**) RCC4 and RCC4/VHL cells were treated with 2 *μ*M CPT, 100 *μ*M etoposide (ETO) or 400 nM of the mTORC1/2 kinase inhibitor pp242 for 24 h. Whole-cell lysates were assayed by western blot for phosphorylated p53 (S15), CHK1 (S317, S345), *γ*H2AX (S139), total p53 and CHK1 proteins. Actin was used as a loading control. (**b**) The 786-O and 786-O/VHL cells were transfected with siRNA to ATR or nonsilencing control (NSC) for 24 h before addition of 2 *μ*M CPT for a further 24 h. mRNA expression of ET-1 was assessed by real-time quantitative PCR relative to GAPDH. Mean±S.E. of duplicate values of one representative experiment is shown. (**c**) RCC4/VHL cells were treated with 10 *μ*M ATM inhibitor (ATMi), 400 nM pp242 or vehicle control (DMSO) in the absence (−) or presence (+) of 2 *μ*M CPT for 24 h. Whole-cell lysates were assayed by western blot for phosphorylated p53 (S15), and p53 proteins. Short and long exposure times are shown for P-S15-p53. Actin was used as a loading control. (**d**) RCC4/VHL and 786-O/VHL cells were preincubated with 10 *μ*M ATMi for 1 h before addition of 2 *μ*M CPT for a further 24 h and mRNA expression of ET-1 was assessed by real-time quantitative PCR relative to GAPDH. Mean±S.E. of duplicate values of one representative experiment is shown

To further explore the DNA damage response in ccRCC cells and the effects on ET-1, we evaluated the canonical ataxia telangiectasia mutated/ataxia telangiectasia and Rad3-related (ATM/ATR) DNA damage pathway. Both ATM and ATR phosphorylate p53 and can signal to CHK-1 and CHK-2 pathways in response to DNA damage.^30^ Bertout *et al.*^13^ have demonstrated that p53 phosphorylation and accumulation following knockdown of HIF-2*α* was prevented by an inhibitor of ATM signaling. In addition, these authors show that radiation-induced CHK-2 phosphorylation was also inhibited by HIF-2*α*, demonstrating HIF-2*α-*mediated suppression of the DNA damage response pathway in ccRCC. Here we also demonstrate inhibition of DNA damage accumulation in RCC4 cells overexpressing both HIF-1*α* and HIF-2*α* in response to chemotherapy (Figure 6a). CPT- and etoposide-mediated phosphorylation of CHK-1 at Ser317 and Ser345 was suppressed in RCC4 cells as compared with VHL-competent cells. CPT-mediated phosphorylation of the histone protein H2AX at Ser139 (*γ*H2AX) was also suppressed in RCC4 cells as compared with their VHL-expressing counterparts (Figure 6a). These results are consistent with a phenotype that implicates HIF-*α* in suppression of DNA damage response proteins in ccRCC. We next investigated the effects of ATR/ATM signaling on p53-mediated regulation of ET-1 and found that inhibition of ATR by siRNA had no effect on CPT-induced ET-1 mRNA levels (Figure 6b), whereas an inhibitor to ATM partially attenuated CPT-mediated ET-1 induction in both 786-O/VHL and RCC4/VHL cell lines and p53 phosphorylation (Figures 6c and d), suggesting that ATM signaling was in part responsible for p53-induced increase in ET-1 mRNA.

### Attenuation of mTORC1/2 signaling suppresses p53-dependent DNA damage responses in ccRCC

**Figure 7:**
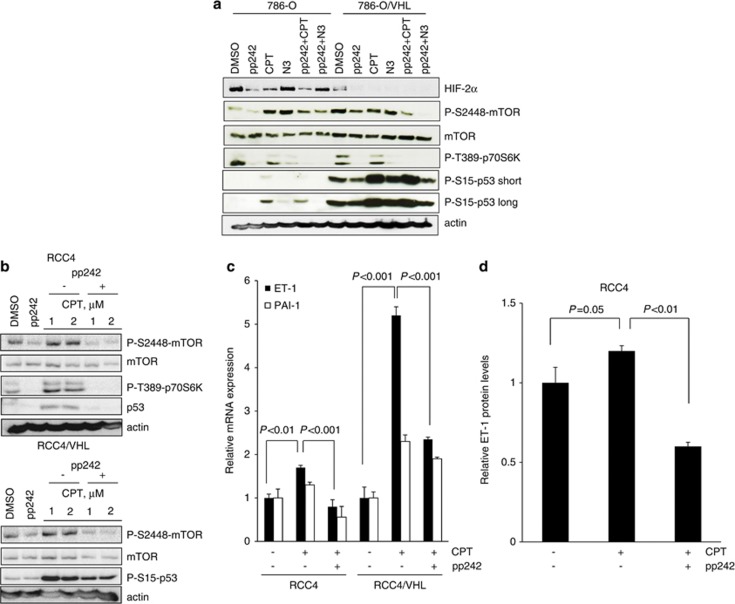
Attenuation of mTORC1/2 kinase ablates CPT-induced p53- and p53-dependent responses in ccRCC. (**a**) The 786-O and 786-O/VHL cells were treated with 400 nM pp242, 2 *μ*M CPT, 10 *μ*M nutlin-3a (N3) or vehicle control (DMSO) alone for 24 h, or preincubated with 400 nM pp242 for 1 h before addition of 2 *μ*M CPT or 10 *μ*M N3 for a further 24 h. Whole-cell lysates were assayed by western blot for HIF-2*α*, mTOR, phosphorylated p53 (S15), mTOR (S2448) and p70S6K (T389) proteins. Actin was used as a loading control. Short and long exposure times are shown for P-S15-p53. (**b**) RCC4 and RCC4/VHL cells were treated with 400 nM pp242 or vehicle control (DMSO) alone or preincubated with 400 nM pp242 for 1 h before addition of either 1 *μ*M or 2 *μ*M CPT for 24 h. Whole-cell lysates were assayed by western blot for mTOR, p53, phosphorylated p53 (S15), mTOR (S2448) and p70S6K (T389) proteins. Actin was used as a loading control. (**c**) RCC4 and RCC4/VHL cells were preincubated with 400 nM pp242 for 1 h before addition of 2 *μ*M CPT for 24 h and mRNA expression of ET-1 and PAI-1 were assessed by real-time quantitative PCR relative to GAPDH. Mean±S.E. of duplicate values of one representative experiment is shown. (**d**) RCC4 cells were pretreated with 400 nM pp242 for 1 h before addition of 2 *μ*M CPT for 24 h. Conditioned media were harvested and assessed for secreted ET-1 protein levels by ELISA and normalized to total protein levels. Mean±S.E. of duplicate values of one representative experiment is shown

**Figure 8:**
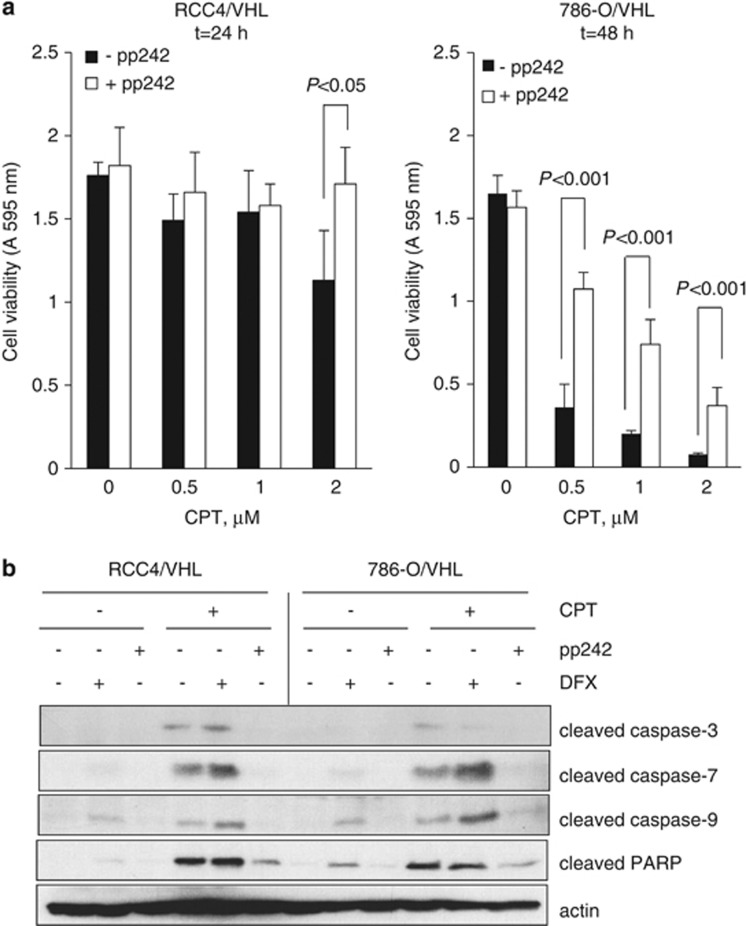
mTORC1/2 kinase inhibition reverses CPT-induced cell death in ccRCC. (**a**) RCC4/VHL and 786-O/VHL cells were incubated with (open bars) or without (solid bars) 400 nM pp242 for 1 h before addition of increasing concentrations of CPT as indicated for 24 or 48 h. Cell viability was determined by reduction of MTT at A 595 nm. Mean±S.E. of triplicate values is shown. (**b**) RCC4/VHL and 786-O/VHL cells were incubated with 400 nM pp242 or 500 *μ*M DFX for 1 h before addition of 2 *μ*M CPT as indicated for 48 h. Whole-cell lysates were assayed for cleaved caspases 3, 7 and 9 and cleaved PARP. Actin was used as a loading control

Inhibitors of mTOR have clinical utility for metastatic ccRCC. The mTOR kinase acts downstream of phosphoinositide 3-kinase (PI3K/Akt) signaling and exists in two multiprotein complexes: mTORC1 and mTORC2. Recent data suggest that the mTORC2 pathway is important for HIF-2*α* translation,^31, 32^ suggesting that dual mTORC1/2 kinase inhibitors may be more beneficial for metastatic ccRCC than rapamycin analogs. We were interested in addressing the question as to whether suppression of HIF-2*α* as mediated by pharmacological blockade of mTORC1/2 kinase activity affected p53 levels in ccRCC. We used the ATP competitive inhibitor of mTOR, pp244, that inhibits both mTORC1 and mTORC2 signaling. pp242 significantly inhibited the phosphorylation of mTOR and its substrate p70S6K and, as expected, it inhibited HIF-2*α* accumulation in 786-O cells (Figure 7a) and both HIF-1*α* and HIF-2*α* in RCC4 cells, consistent with previous studies (Supplementary Figure 4A).^31, 33^ Rapamycin, which suppresses mTORC1 signaling, did not affect HIF-2*α* protein levels, confirming a role for mTORC2 in the regulation of HIF-2*α* (Supplementary Figure 4B). Interestingly, rapamycin also had little effect on HIF-1*α* accumulation (Supplementary Figure 4B). Next, we assessed the effects of pp242 on p53 levels. Treatment with pp242 did not increase p53 phosphorylation or accumulation as might be predicted from the downregulation of HIF-2*α* in 786-O cells (Figure 7a) and RCC4 cells (Supplementary Figure 4A). This suggests that either low levels of HIF-2*α* are sufficient for effective suppression of p53 or that mTOR is required for p53 accumulation in ccRCC. In order to address the latter, we assessed p53 levels in response to pp242. Pharmacological inhibition of mTOR with pp242 attenuated p53 phosphorylation and accumulation in response to CPT (Figure 7 and Supplementary Figure 5). We confirmed that the effects of pp242 on p53 phosphorylation were not due to a nonspecific inhibition of ATM kinase activity as the mTORC1/2 kinase inhibitor had no effect on phosphorylation levels of ATM, whereas it markedly reduced CPT-mediated p53 phosphorylation in ccRCC cell lines (Supplementary Figure 5A). In addition, no inhibition of ATM phosphorylation by pp242 was observed in MCF-7 and HEK-293 cells following DNA damage with either ultraviolet irradiation or etoposide treatment (Supplementary Figures 5B and C). Moreover, specific targeting of mTOR with siRNA also markedly suppressed p53 phosphorylation in HEK-293 cells (Supplementary Figure 5D). We next assessed the ability of pp242 to inhibit p53-dependent responses in ccRCC; CPT-dependent induction of PAI-1 was partially inhibited by pp242 treatment (Figure 7c). Consistent with a role for p53 in the regulation of ET-1, pp242 significantly attenuated CPT-dependent increase in ET-1 mRNA abundance in RCC4 and RCC4/VHL cells (Figure 7c) and secreted protein levels in RCC4 cells (Figure 7d). Moreover, CPT-dependent cell death was strikingly inhibited by pp242 in ccRCC cell lines (Figure 8a), as was CPT-induced cleavage of caspases 3, 7 and 9 as well as PARP cleavage (Figure 8b). Taken together, these results suggest a requirement of mTOR for p53-mediated apoptosis in ccRCC.

## Discussion

There has been a renewed interest in assessing the DNA-damaging agent CPT for anticancer therapy in ccRCC and other solid tumors as it possesses significant HIF-1*α* inhibitory activity. Early clinical studies of CPT and its analogs, topotecan and irinotecan, failed to show activity in clinical trials for renal cancer as patients suffered severe toxicity that limited their clinical utility.^34, 35^ Recent efforts to circumvent the toxicity observed with CPT have led to the development of novel delivery systems such as the cyclodextrin-containing polymer nanopharmaceutical, CRLX101, that has improved solubility and retention profiles.^36^ CRLX101 is currently in clinical trials as a single agent for a range of tumor types and combined with Avastin for advanced kidney cancer.

As topoisomerase I inhibitors are potent inhibitors of HIF-1*α*, we were interested in whether CPT also inhibited HIF-2*α*, which is considered to be the more important subunit for kidney cancer progression. Here we addressed the effects of CPT on HIF-*α* expression and activity and p53-dependent responses. CPT ablated both HIF-1*α* and HIF-2*α* but failed to significantly inhibit HIF-2*α* target genes in cells that express both HIF-*α* subunits. Instead, CPT increased apoptosis in VHL-defective ccRCC cell lines that was augmented in their VHL-expressing counterparts, confirming a role for the VHL/HIF pathway in resistance to chemotherapeutics consistent with previous studies.^13, 14, 24, 26^  We still observed some resistance to p53-dependent apoptosis in 786-O cells with CPT as compared with 786-O/VHL cells despite concurrent inhibition of HIF-2*α*, suggesting that low levels of HIF-2*α* are able to effectively suppress p53, or that other HIF-independent VHL pathways are also operative.^26^ In this study we did not attempt to address the mechanism by which the VHL/HIF pathway affects p53 as this has been studied extensively by other authors.^13, 14, 24, 26, 37^ Instead, we were concerned with the downstream outcome of chemotherapy treatment that could concurrently inhibit HIF-*α* and activate the p53 pathway.

p53 regulates cellular responses to stress that include the ability to induce cell cycle arrest, apoptosis and senescence through activating p53-responsive genes.^38^ Of interest was the increased secreted phenotype in RCC4 cells treated with high concentrations of CPT. Previous studies have demonstrated a senescence-associated secretory phenotype (SASP) upon severe DNA damage accompanied with increased p53, p21^CIP1^ and PAI-1 levels.^39^ In RCC4 cells, CPT increased p53 accumulation and activated the p53 target genes, p21 and cyclin D1, and increased secretion of VEGF, PAI-1 and ET-1 that have all been associated with senescence in other cell types.^40, 41, 42, 43^ In addition to HIF-*α* regulation, PAI-1 is also a p53 target gene and we could show that PAI-1 mRNA was increased in a p53-dependent manner on CPT treatment. Our data suggested that ET-1 was regulated in a similar manner to PAI-1, and indeed we could show that increased ET-1 levels were p53 dependent upon DNA damage, whereas constitutive ET-1 mRNA and protein levels were primarily dependent on VHL/HIF. To determine whether p53 might regulate ET-1 transcription directly, we performed a search of the proximal promoter, and three putative p53 DNA-binding sites were identified (Supplementary Figure 6A). One of these, designated DNA-binding site 1 (DBS1), has high homology with mouse and rat ET-1 genes. Interestingly, a recent study has demonstrated ET-1 to be a transcriptional target of p53 in epidermal keratinocytes, raising the possibility that p53 may regulate ET-1 transcription directly.^44^

In the vascular system, ET-1 is a potent vasoconstrictor and has a diverse range of physiological functions including maintenance of vessel tone, smooth muscle cell proliferation and promotion of angiogenesis.^45^ Pathophysiological levels of ET-1 contribute to a number of cardiovascular diseases.^46, 47, 48^ In the kidney, it has been reported that ET-1 is a survival factor for renal carcinoma cells that express the ET-A receptor (ET_A_),^49^ and a recent report suggested that patients receiving ET receptor antagonist therapy combined with interferon-*α* had a better outcome than interferon-*α* alone,^50^ suggesting that blockade of ET-1 signaling would be beneficial for metastatic RCC. In this context, p53-dependent increase of ET-1 may limit efficacy of chemotherapeutics; however, we found little effect of ET-A receptor blockade on CPT-mediated cell death (Supplementary Figures 6C and D). Nevertheless, the biological significance of the p53-dependent increase of ET-1 warrants further investigation.

The mTOR pathway is a major cancer drug target and renal cell carcinoma is one cancer that has demonstrated clinical success with rapalog therapy. mTOR exists in two multiprotein functional complexes: mTOR complex 1 (mTORC1) and mTOR complex 2 (mTORC2). Rapamycin, which binds the FKBP-rapamycin-binding domain and not the ATP binding pocket, predominantly inhibits mTORC1 complex formation.^51^ As recent data have demonstrated that mTORC2 is important for HIF-2*α* protein translation in ccRCC,^31, 32^ we asked the question of whether HIF-2*α* ablation by an mTORC1/2 kinase inhibitor, which interacts with the ATP binding pocket of mTOR and thus inhibits both mTORC1 and mTORC2 activity, affected p53 status in ccRCC cells. We could show that HIF-2*α* inhibition mediated by pp242 did not result in increased p53 accumulation. Instead, mTOR was required for p53 phosphorylation and accumulation following CPT treatment in both 786-O and RCC4 cells and their VHL-expressing counterparts. mTOR and p53 pathways act in a coordinated manner to regulate cell growth, proliferation and death depending on extracellular signals. p53 can attenuate mTOR activity and signaling in response to genotoxic stress by induction of p53 target genes.^52, 53^ In addition, in other cellular contexts, mTOR can regulate p53.^54^ Our results are consistent with the latter observations.

Collectively, our data suggest that CPT is unlikely to mediate its antitumor effects by inhibition of HIF-2*α* target genes such as VEGF and support current clinical efforts combining Avastin with CPT-based nanodrugs. Importantly, we show that CPT mediates its effects through classical DNA damage-induced p53-dependent apoptosis. Moreover, we uncover a novel regulation of ET-1 by p53 in ccRCC and show that p53-dependent apoptosis is dependent on mTORC1/2 kinase activity in this tumor type, suggesting that potential combination therapies of chemotherapeutics with mTOR inhibitors need to be carefully evaluated for ccRCC.

## Materials and Methods

CPT, nutlin-3a, etoposide, apigenin, desferrioxamine, cycloheximide, MG-132, methylthiazoletetrazolium (MTT) and pp242 were all purchased from Sigma-Aldrich (Gillingham, UK). The ATM kinase inhibitor was obtained from Calbiochem (Millipore, Watford, UK).

### Cell culture

All tumor cell lines were maintained in Dulbecco's modified Eagle's medium (PAA Laboratories, Yeovil, UK) supplemented with 10% fetal bovine serum (Sigma-Aldrich), 100 IU/ml penicillin, 100 *μ*g/ml streptomycin and 2 mM glutamine (all purchased from Life Technologies, Paisley, UK). Matched RCC4 renal cell carcinoma cells (RCC4 and RCC4/VHL) were gifts from Professor Patrick Maxwell (Cambridge University, UK).^55^ The 786-O renal cell carcinoma cells (786-O and 786-O/VHL) were gifts from Professor William G. Kaelin Jr (Dana-Farber Cancer Institute, Harvard Medical School, Boston, MA, USA). Matched human colorectal carcinoma cells (HCT-116 p53^+/+^ and HCT p53^−/−^) were kindly provided by Professor Galina Selivanova (Karolinska Institute, Stockholm, Sweden). The p53-null Saos-2 cells were obtained from American Type Culture Collection (Manassas, VA, USA).

### Immunoblotting

Cell treatments were performed in DMEM media containing 10% FBS for the times and concentrations as indicated in the figure legends. Thereafter, conditioned medium was harvested and analyzed by ELISA for PAI-1 (IMUBIND, American Diagnostica, Axis-Shield, Stockport, UK), VEGF (QuantiGlo, R&D Systems, Abingdon, UK) and ET-1 (Quantikine, R&D Systems). In parallel, whole-cell lysates were prepared for western blotting as described previously.^56^ HIF-1*α* monoclonal antibody was purchased from BD Biosciences (Oxford, UK). HIF-2*α* polyclonal antibody (NB100-122) was purchased from Novus Biologicals (Cambridge, UK). Monoclonal anti-actin antibody was purchased from Merck (Nottingham, UK) and anti-*α*-tubulin antibody was obtained from Sigma. Monoclonal anti-CHK-1 antibody (sc-8408) was purchased from Santa Cruz (Santa Cruz, CA, USA). Monoclonal anti-cyclin D1 (DCS-6) was purchased from Thermo Fisher Scientific (Runcorn, UK). Monoclonal anti-phospho-S15-p53, and the polyclonal-anti-phospho-antibodies S2448-mTOR, S1981-ATM, T389-p70S6K, S317-CHK-1, S296-CHK-1, S345-CHK-1, S139-*γ*H2AX and total p53, mTOR, cleaved PARP and cleaved caspase 3, 7 and 9 antibodies were all obtained from Cell Signaling (New England Biolabs, Hitchin, UK).

### siRNA duplexes and transient transfections

Cells were transfected with siRNA duplexes using HiPerfect (QIAGEN, Crawley, UK) transfection reagent according to the manufacturer's instructions and as described previously.^9^ siRNA duplexes to HIF-1*α*, HIF-2*α*, p53 and a non-silencing control (NSC) duplex were obtained from QIAGEN and have been described previously.^9, 57^ ATR siRNA (sc-29763) was purchased from Santa Cruz. AllStars negative control siRNA duplex was purchased from QIAGEN.

### Real-time quantitative PCR assays

Total RNA was extracted from cells using the RNeasy Mini Kit (QIAGEN) and 50 ng total RNA was used for first-strand cDNA synthesis using Sensiscript (QIAGEN) according to the manufacturer's instructions. Real-time qPCR was performed with GoTaq qPCR master mix (Promega, Southampton, UK) in a Stratagene Mx3000 real time cycler (Agilent Technologies, Stockport, UK). Primers for ET-1 were purchased from Sigma-Aldrich and have been described previously (forward 5′-TGGACATCATTTGGGTCAACA; reverse 5′-TCTCTTGGACCTAGGGCTTCC).^58^ Primers for PAI-1, HIF-2*α* and GAPDH have been described previously.^9^ Expression of target gene mRNA relative to reference gene mRNA (GAPDH) was calculated using the Relative Expression Software Tool (REST, QIAGEN) and statistical significance determined using the Pair Wise Fixed Reallocation Randomization Test available from http://www.gene-quantification.com.

### Flow cytometry

Flow cytometry was performed as previously described.^56^ Briefly, following treatment, cells were harvested and re-suspended in 70% ethanol. Cells were washed with PBS and resuspended in 50 *μ*g/ml propidium iodide/RNAse A solution (Sigma-Aldrich) and analyzed with a Beckman Coulter Cytomics FC500 flow cytometer.

### MTT cell viability assay

Cell viability was determined by reduction of MTT (Sigma-Aldrich). Briefly, cells were seeded in 96-well plates, washed with PBS before addition of test compounds. MTT reagent (25 *μ*l) was added to wells after 24 or 48 h and incubated for a further 2 h. Thereafter, 100 *μ*l of 10% SDS was added to wells and incubated for 24 h before measurement of absorbance at 595 nm.

## Abbreviations

mTORC1/2: mammalian target of rapamycin complex 1 and complex 2
HIF: hypoxia-inducible factor
ET-1: endothelin-1
ccRCC: clear cell renal cell carcinoma
CPT: camptothecin
VEGF: vascular endothelial growth factor
PAI-1: plasminogen activator inhibitor-1
VHL: von Hippel–Lindau
ATM: ataxia telangiectasia mutated
ATR: ataxia telangiectasia and Rad3-related
PDGF: platelet-derived growth factor
PARP: poly ADP ribose polymerase
