# Supplementary material for: Chemotherapy-mediated p53-dependent DNA damage response in clear cell renal cell carcinoma: role of the mTORC1/2 and hypoxia-inducible factor pathways
Source: Cell Death Dis. 2013 Oct 17;4(10):e865–. doi: 10.1038/cddis.2013.395 (PMC3920935; doi:10.1038/cddis.2013.395)

Supplementary Fig 1

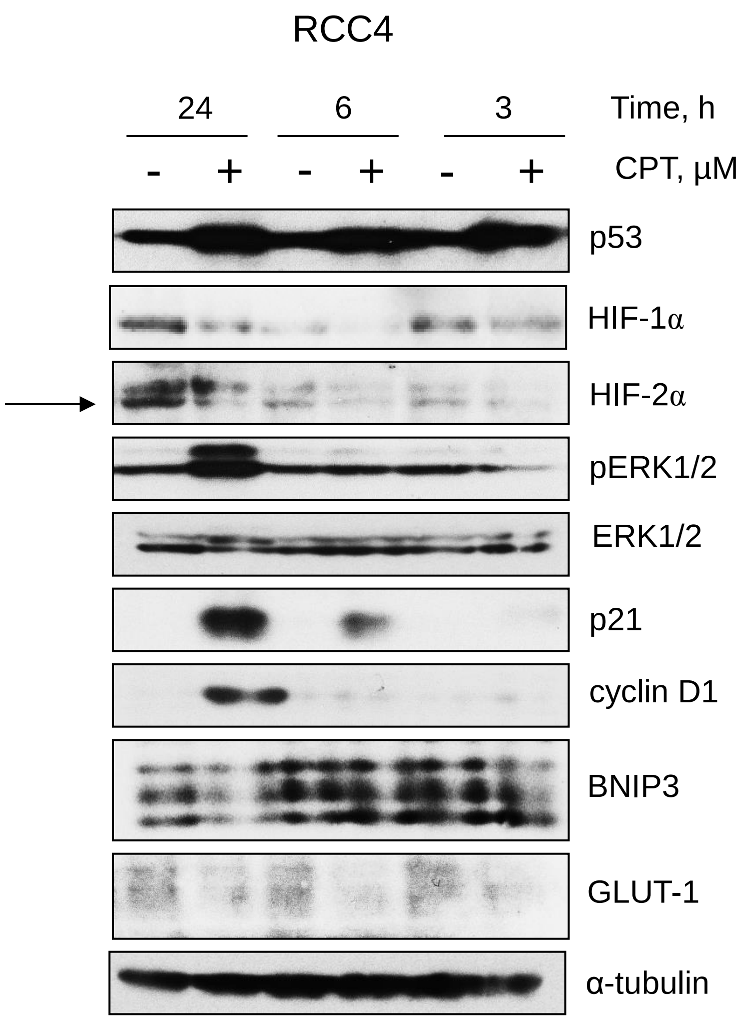

Supplementary Fig 2

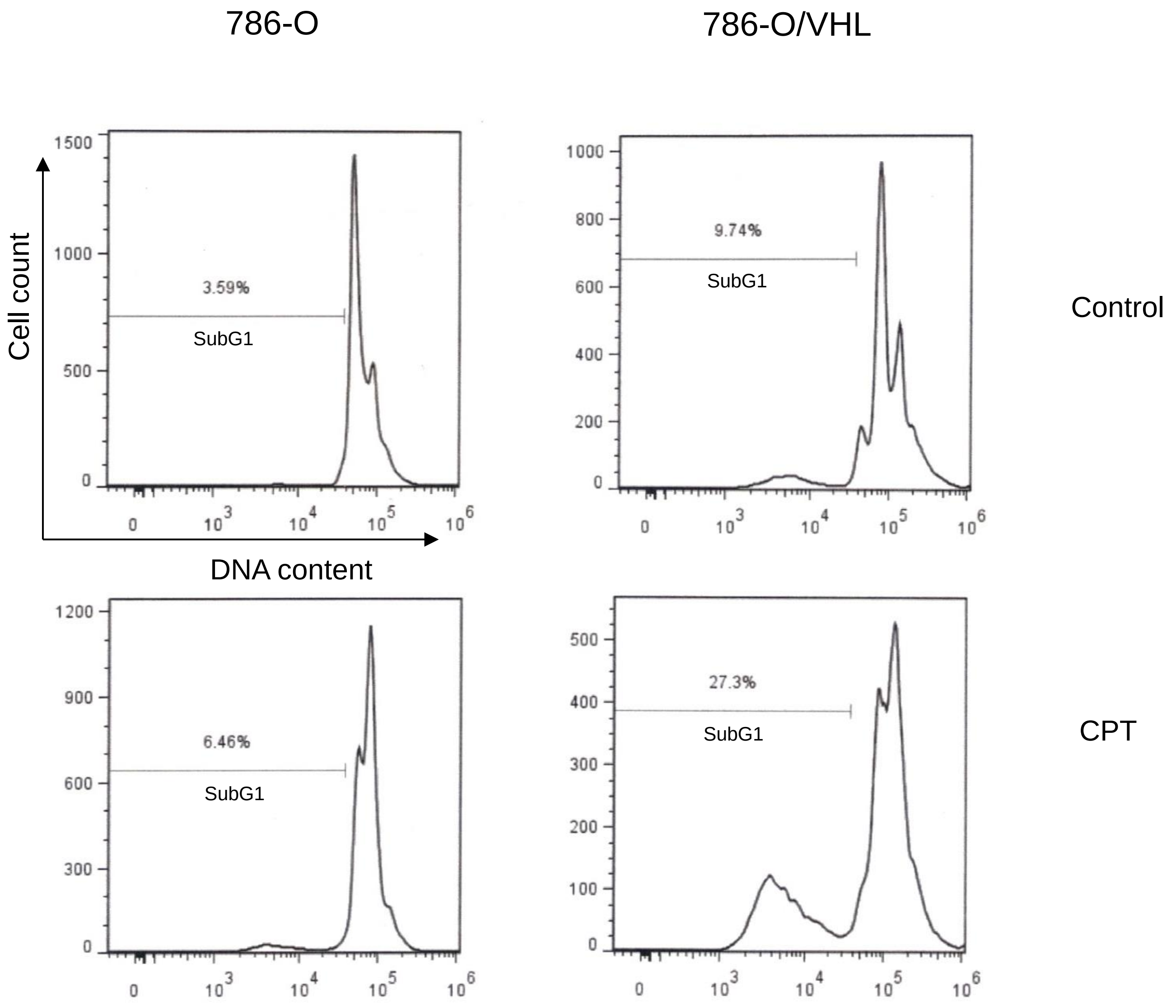

Supplementary Fig 3

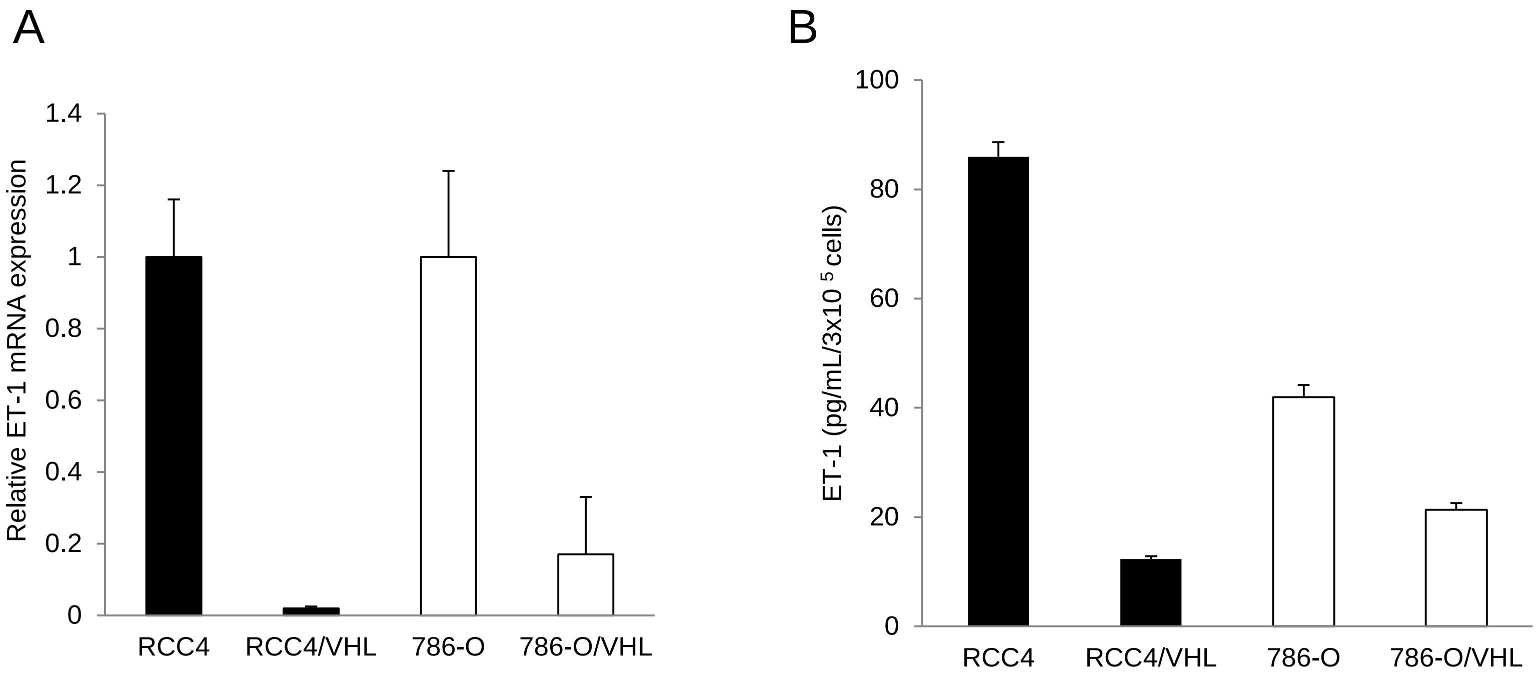

Supplementary Fig 4

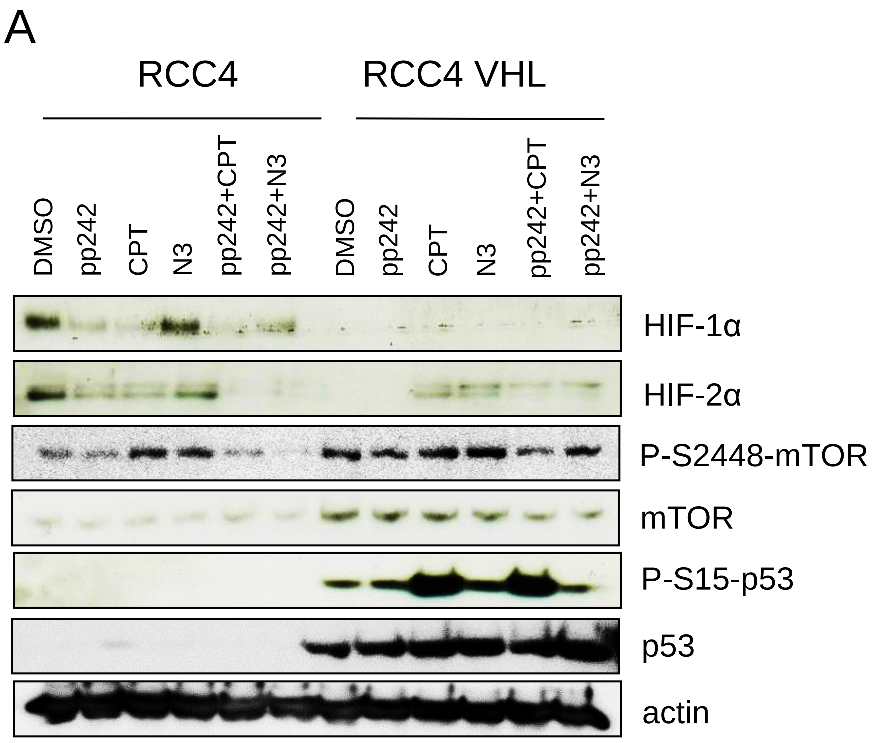

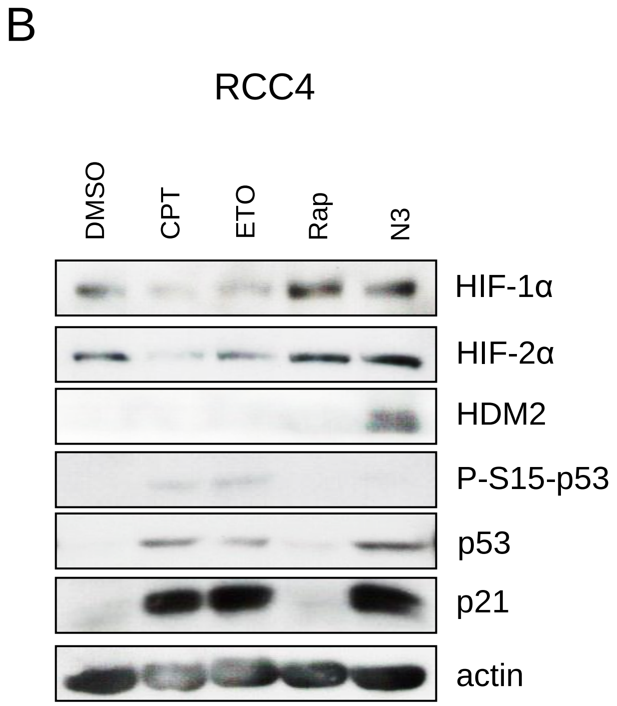

Supplementary Fig 5

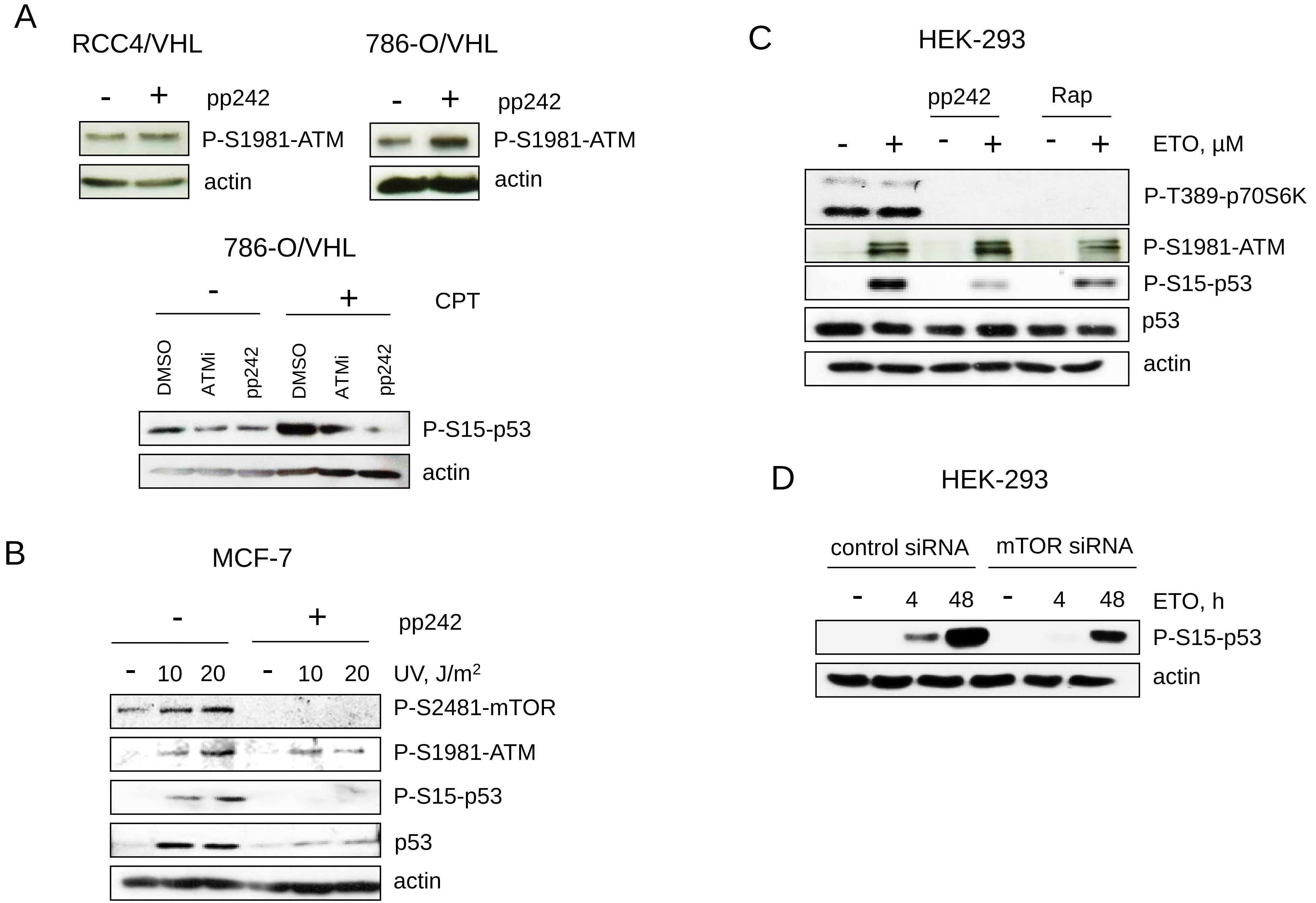

Supplementary Fig 6

A

```
                                        p53 DBS3(-1575 to -1597)
421   ggctctttta gaggatgcat gtcatta tgg acctgtcgct gccactgtcc ctgttccccc
481   agctgtgact tcgagggagg tctggggatc tgagtctgtc caaacccacg gctttgctgt
541   tgggataaaa actgtccttt tgattttaga aggaggaggg aaaaaaggtt tcccagcatg
601   tgtgttgtgc cagtcttgga aattcatccg tgcttgaatt ccaccctcca tccccagaaa
661   aactggagta aaacaaaaag aggagatgga caaagtgtgt atttgatggc atcccctggg
721   aagagactct aaatttatcc cataggtctt actgggccac tgtgagcgct ttggtggaga
781   acaaacaaaa attctgggtg ctcagttgtc taacctgaaa aatgggacta gcggaaaaag
841   ccaatgtgtt ccatgcacct tttgctttct ttattaaggc atgatgtcac ctgtacagta
901   actgccctgt gtgtacttca gggggggatt tcaaggttag atagacagga aattgttttg
961   aaaatgtaaa cacattatta aatgtgaagt attatctgat tccttgttcg aatggcattt
1021  ccttctcagc accaccttcc ttgcatattc acttaacctt gtacaagaac acctttttgc
1081  cctaaatgaa gacacccccc caaaaaaaag agtcccagaa aatatgtccc tgcttgtgcg
1141  gggaataaat agaatattct gaggtgcatt cctccttcct atgttaggca acattccttg
1201  accctcctcg gcccccaagc caggttgcgt tttttctgc catttagaag ggttttcctt
1261  tttgtcctag taaaacatca gcccctgtag ctcttcatct ccccctggtg ttcttctccc
        p53 DBS2(-704 to -726)
1321  gccatgtctt aagattggtg gcaccgacca atcttaagat ttaagttctg tgtgaaaaac
1381  acctttgctt ttcaatcagt ttatcagcct cctccgcagg ggaagtgtgg acacacaaaa
1441  gaacttatcg gggcttctca tcagtgatag ggaaaagact ggcatgtgcc taaacgagct
1501  ctgatgttat ttttaagctc cctttcttgc caatccctca cggatctttc tccgatagat
1561  gcaaagaact tcagcaaaaa agacccgcag gaaggggctt gaagagaaaa gtacgttgat
1621  ctgccaaaat agtctgaccc ccagtagtgg ggcagtgacg agggagagca ttcccttgtt
1681  tgactgagac tagaatcgga gagacataaa aggaaaatga agcgagcaac aattaaaaaa
1741  aattccccgc acacaacaat acaatctatt taaactgtgg ctcatacttt tcataccaat
1801  ggtatgactt tttttctgga gtcccctctt ctgattcttg aactccgggg ctggcagctt
1861  gcaaagggga agcggactcc agcactgcac gggcaggttt agcaaaggtc tctaatgggt
                  p53 DBS1(-189 to -211)
1921  attttctttt tcttagccct gcccccgaat tgtcagacgg cgggcgtctg cctctgaagt
                                          HRE(-118)
1981  tagcagtgat ttcctttcgg gcctggcctt atctccggct gcacgttgcc tgttggtgac
2041  taataacaca ataacattgt ctggggctgg aataaagtcg gagctgttta cccccactct

                                                 +1
2101  aataggggtt caatataaaa agccggcaga gagctgtcca agtcagacgc gcctctgcat
                                               →start
```

B

human	T	A G C C C T G C C C	C c g a A T T G T C	A G
rat	T	A G C C C T G C C C	C T G G A T T G T C	A G
mouse	T	A G C C C T G C C C	C T G G A T T G T C	A G

Supplementary Fig 6

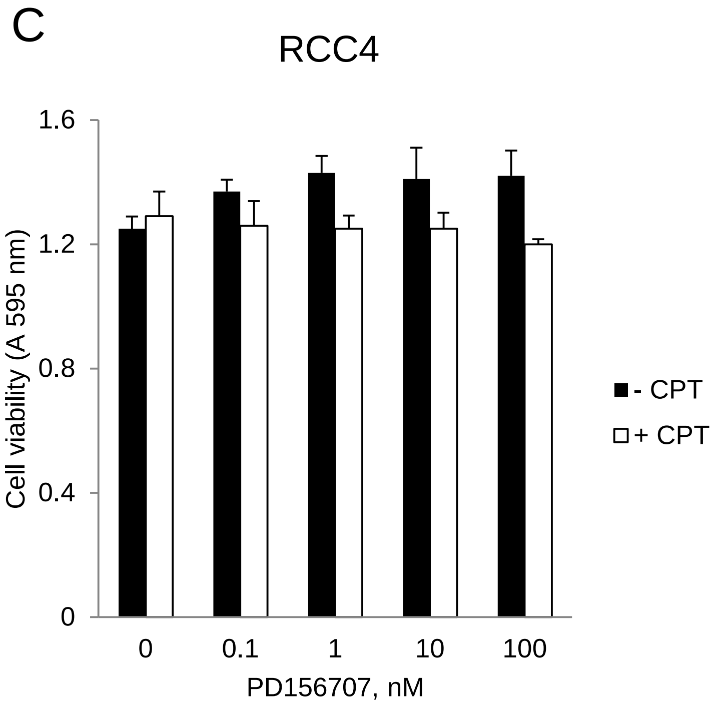

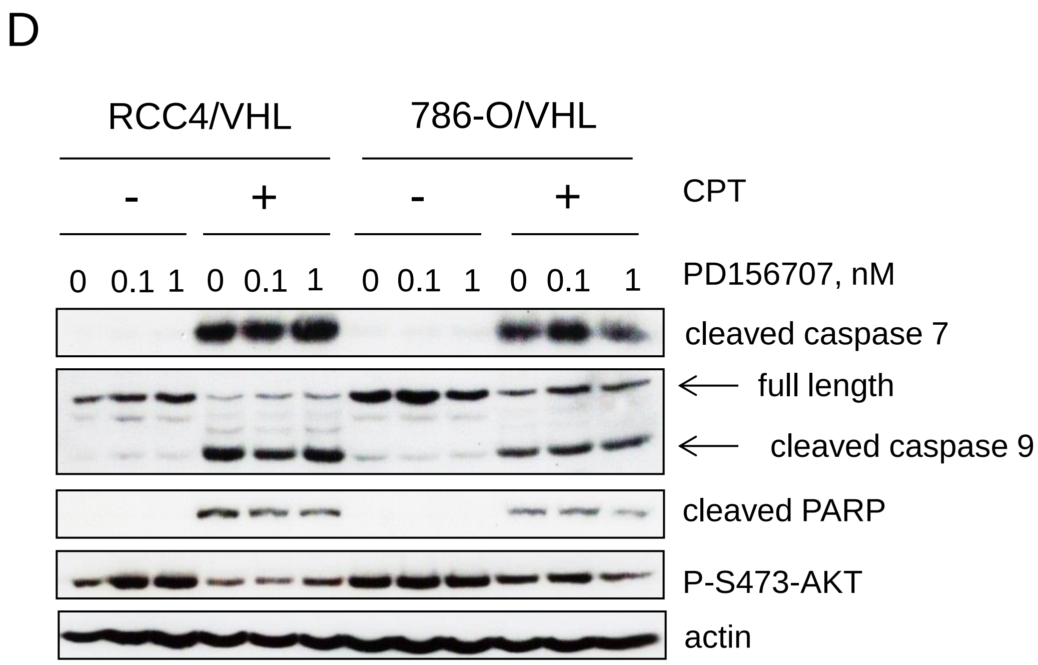

Supplement: Supplementary Figures [file cddis2013395x1.pdf]
