# Supplementary material for: Chemotherapy-mediated p53-dependent DNA damage response in clear cell renal cell carcinoma: role of the mTORC1/2 and hypoxia-inducible factor pathways
Source: Cell Death Dis. 2013 Oct 17;4(10):e865–. doi: 10.1038/cddis.2013.395 (PMC3920935; doi:10.1038/cddis.2013.395)
Supplement: Supplementary Information [file cddis2013395x2.doc]

**Supplementary Text**

**Materials**

Rapamycin and monoclonal anti-BNIP3 (Ana40) antibody were purchased from Sigma. mTOR siRNA, polyclonal anti-phospho-p44/42 MAPK (pERK1/2) and anti-phospho-S473-AKT antibodies were obtained from Cell Signaling (NEB, Hitchin, UK). Polyclonal anti-p21 (sc-397) and anti-ERK antibodies and monoclonal anti-HDM2 (SC965) antibody were all purchased from Santa Cruz, CA, USA. Polyclonal GLUT-1 antibody was purchased from Abcam, Cambridge, UK. ET-A receptor antagonist, PD156707 was obtained from R&D Systems. MCF-7 and HEK-293 cell lines were purchased from American Type Culture Collection (Manassas, VA, USA).

**Figure Legends**

**Figure 1. Effect of CPT on HIF-α accumulation and HIF-α target genes**

RCC4 cells were treated with 2 µM CPT (+) or DMSO vehicle control (-) for the times indicated. Whole-cell lysates were assayed by Western blot for HIF-1α, HIF-2α, p53, p21, cyclin D1, BNIP3, GLUT-1, phosphorylated p44/p42 MAPK (pERK1/2) and total ERK1/2 proteins. Tubulin was used as a loading control.

**Figure 2. CPT mediated apoptosis is augmented in VHL competent cells**

786-O and 786-O/VHL cells were treated with 100 nM CPT or vehicle for 24 h, fixed, stained with propidium iodide and the percentage (%) of cells in subG1 was assessed by flow cytometry. Histogram plots for representative samples are shown.

**Figure 3. ET-1 mRNA and protein is regulated by pVHL in ccRCC**

*A*, RCC4 and 786-O cells and their VHL expressing counterparts were assayed for mRNA expression of ET-1 by real-time quantitative PCR relative to GAPDH. *B*, RCC4 and 786-O cells and their VHL expressing counterparts were assayed for ET-1 protein levels at 24 h and normalised to cell number. Mean ± SE of duplicate values of one representative experiment is shown.

**Figure 4. Effect of mTOR inhibitors on HIF-α and p53 accumulation**

*A*, RCC4 and RCC4/VHL cells were treated with 400 nM pp242, 2 µM CPT, 10 µM nutlin-3a (N3) or vehicle control (DMSO) alone for 24 h, or preincubated with 400 nM pp242 for 1 h before addition of 2 µM CPT or 10 µM nutlin-3a (N3) for a further 24 h. Whole cell lysates were assayed by Western blot for HIF-1α, HIF-2α, mTOR, and p53 proteins and phosphorylated p53 (S15) and mTOR (S2448) proteins. Actin was used as a loading control. *B*, RCC4 cells were treated with 2 µM CPT, 100 µM etoposide (ETO), 200 nM rapamycin (Rap),10 µM nutlin-3a (N3) or vehicle control (DMSO) for 24 h. Whole cell lysates were assayed by Western blot for HIF-1α, HIF-2α, HDM2, p53 and p21 proteins and phosphorylated p53 (S15). Actin was used as a loading control.

**Figure 5. Pharmacological inhibition of mTORC1/2 has no effect on ATM kinase phosphorylation**

*A*, RCC4/VHL and 786-O/VHL cells were incubated with or without 400 nM pp242, 10 µM ATM inhibitor (ATMi) or vehicle control (DMSO) in the absence or presence of 2 µM CPT for 24 h. Whole-cell lysates were assayed by Western blot for phosphorylated ATM (S1981) and p53 (S15). Actin was used as a loading control. *B*, MCF-7 cells were exposed to ultraviolet (UV) radiation as indicated before addition of 400 nM pp242 for 4 h. Whole-cell lysates were assayed by Western blot for phosphorylated mTOR (S2481), ATM (S1981), p53 (S15) and total p53 protein. Actin was used as a loading control. *C*, HEK-293 cells were incubated with 400 nM pp242 or 200 nM rapamycin (Rap) for 1 h before addition of 100 µM etoposide (ETO) as indicated for 4 h. Whole-cell lysates were assayed by Western blot for phosphorylated p70S6K (T389), ATM (S1981), p53 (S15) and total p53 protein. Actin was used as a loading control. *D*, HEK-293 cells were incubated with 25 nM mTOR siRNA or control duplex for 24 h before addition of 100 µM etoposide (ETO) for the times indicated. Whole-cell lysates were assayed by Western blot for phosphorylated p53 (S15). Actin was used as a loading control.

**Figure 6. Identification of three putative p53 response elements in the ET-1 proximal promoter**

*A,* The promoter region of human *ET-1* (edn1) gene from position – 1624 to + 16 relative to the transcription start site is shown. Three putative p53 DNA binding sites (DBS) are highlighted. All three putative p53 DBS are in the reverse orientation as is the hypoxia response element (HRE) which is also shown. *B*, Alignment of the human *ET-1* putative p53 DNA binding site 1 with rat and mouse genes. C, RCC4 cells were incubated with increasing concentrations of the ET-A receptor antagonist, PD156707, as indicated for 1 h before addition of 2 µM CPT (open bars) or vehicle control (solid bars) for 24 h. Cell viability was determined by reduction of MTT at A 595 nm. Mean ± SE of triplicate values is shown. *D,* RCC4/VHL and 786-O/VHL cells were incubated with increasing concentrations of PD156707 as indicated for 1 h before addition of 2 µM CPT for 24 h. Whole-cell lysates were assayed by Western blot for cleaved caspases 7 and 9, cleaved PARP and phosphorylated AKT (S473). Actin was used as a loading control.
